# Analysis of C-Kit Exon 9, Exon 11 and BRAFV600E Mutations Using Sangers Sequencing in Gastrointestinal Stromal Tumours

**DOI:** 10.7759/cureus.7369

**Published:** 2020-03-22

**Authors:** Divya Madhala, Sandhya Sundaram, MohanaPriya Chinambedudandapani, Archana Balasubramanian

**Affiliations:** 1 Pathology, Sri Ramachandra Institute of Higher Education and Research, Chennai, IND; 2 Genetics, Sri Ramachandra Institute of Higher Education and Research, Chennai, IND

**Keywords:** c-kitmutations, braf v600e mutations, dna sequencing, gastrointestinal stromal tumors

## Abstract

Background

Gastrointestinal stromal tumors (GIST) are the most common mesenchymal neoplasms in the gastrointestinal (GI) tract. The mutation of C-KIT is considered to be the crucial step in the tumorigenesis. Targeted therapies are being developed focusing these mutations. Various exon mutations of GIST responded in varied patterns to this targeted therapy. This study was carried out to evaluate the C-KIT exon 11, exon 9 and BRAF V600E mutations among GIST specimens.

Methods

This retrospective study was carried out among 20 DNA extracted specimens from paraffin blocks of GIST received in our tertiary teaching institution for a period of three years. DNA sequencing was carried out for mutational analyses on C-KIT exon 9, C-KIT exon 11 and BRAF V600E genes using Sanger sequencing.

Results

Histologically, majority of the tumors had spindle cell morphology. About 19 cases were positive for CD117. The analysis of type of mutations showed that three cases carried Exon 11 and three cases carried Exon 9 mutations. BRAF V600E mutation was seen in one case.

Conclusion

It is essential to conduct molecular studies on GISTs in order to get a clear understanding of the pathogenesis and behavior pattern. This will also help in designing targeted therapies and assessing recurrence. With the advent of rapidly evolving personalized therapy, the evaluation of genetic mutations is essential for diagnosis and prognostic value.

## Introduction

Gastrointestinal stromal tumours (GISTs) are the most common mesenchymal neoplasms in the gastrointestinal (GI) tract. They account for < 1% of all gastrointestinal tumours and 5% of all sarcomas. GISTs were initially thought to be rare. However, because of an increased ability to reliably diagnose them, their incidence is now estimated to be around 5000 new cases per year in the United States. No supportive data is available from India in this regard.

The spectrum of GIST varies based on the clinical presentation, location, histology and prognostic outcomes. Although GIST can occur throughout the GI tract, it is increasingly common in the stomach and small bowel, compared to colon, rectum and esophagus. In certain situations, an extra gastrointestinal site involvement is also encountered (EGIST).

The cellular origin of gastrointestinal stromal tumours is the pluripotent mesenchymal stem cell programmed to differentiate into Interstitial Cells of Cajal (ICC) [1]. GISTs show many morphologic, immunohistochemical and molecular features common with ICC [2].

The clinical importance of GIST is its characteristic molecular feature of gain of function mutations in the c-kit proto-oncogenes. This mutation is considered to be the crucial step in the tumorigenesis of GISTs and are found in the smallest sub-centimeter of GIST [3]. The c-KIT mutations are scattered along hotspots including exon 9, exon 11, exon 13 and exon 17 of the c-kit gene. Approximately 67% of the KIT mutations involve exon 11, while 10% involve exon 9 and 1% each in exon 13 and 17. However, rare KIT mutations involve exon 8, 12, 14 and 18. Further molecular analyses showed molecular changes in Platelet Derived Growth Factor Receptor Alpha (PDGFRA), B-Raf (BRAFV600E), Succinate Dehydrogenase (SDH) subunits A, B, C, D and Neurofibromatosis type 1 (NF1) genes.

Also, 12-25% of the GISTs lack c-KIT or PDGFRA mutations; these have been identified as wild type GIST. BRAFV600E mutations have been identified in these wild type GIST and account for 3% of all GISTs [4]. The mutations in GISTs showed varied clinical manifestations and prognostic outcomes.

The clinical significance of c-KIT mutations was the development of targeted therapies at molecular level. The initial drug discovered for the treatment of GISTs was imatinib mesylate, a tyrosine kinase inhibitor. This drug acts by competing for the ATP binding site on the target kinase, thereby inhibiting tyrosine kinase and reducing cellular proliferation [5]. With further knowledge in the GISTs, many tyrosine kinase inhibitors were discovered and Sunitinib was found to be suitable for patients with C-Kit mutations with exon 9 involvement. This warranted the need for evaluating the KIT mutations at the exon level, so as to achieve targeted pharmacotherapy.

Although several studies are available on mutational analysis of GISTs, very few studies have been documented in India. In our study, a mutational analysis of C- KIT exon 9, exon 11 and BRAFV600E mutations were carried out in the Indian population. This will prove useful in designing therapies to target specific mutations and provide better therapeutic outcomes.

## Materials and methods

This retrospective study was carried out on immunohistochemically proven GIST specimens using DNA extracted from paraffin blocks of all the cases received in the Department of Pathology at our tertiary teaching institution from October 2013 to October 2016. A total of 20 proven cases of GIST were taken up for this study. Permission from the Institutional Ethics Committee was obtained prior to commencing the study(IECNO:CSPMED/15/OCT/25/56).

A structured proforma was used to obtain clinical and demographic information of the patients from the medical records. The formalin fixed paraffin embedded tissues were taken up as 4-5 micron sections and stained with hematoxylin and eosin and graded accordingly. One representative block was selected and immunohistochemically stained for c-KIT. The antibody used was mouse monoclonal antibody (CD117) procured from BioGenex Laboratories Inc.

The steps carried out for mutational analyses on c-KIT exon 9, c-KIT exon 11 and BRAF V600E genes include DNA isolation, assessment of DNA quality and quantity using Hybrid Reader (nanodrop), polymerase chain reaction using forward (F) and reverse(R) primers [Table 1], agarose gel electrophoresis and cycle sequencing.

**Table 1:** Forward and Reverse Primers used in PCR for C-KIT Exon 11, Exon 9 and BRAF V600E

Gene	Primer sequence	BP
c-KIT Exon 11(F)	GTGCTCTAATGACTGAGAC	19
c-KIT Exon 11(R)	TACCCAAAAAGGTGACATGG	20
c-KIT Exon 9(F)	CTAGAGTAAGCCAGGGCTTTTGTT	24
c-KIT Exon 9(R)	CCTAAACATCCCCTTAAATTGGATT	25
BRAF(F)	TCATAATGCTTGCTCTGATAGG	22
BRAF(R)	GGCCAAAAATTTAATCAGTGG	21

Following this, data analysis was carried out using the Mutation Taster Software. Majority of the participants belonged to the age group of 50-59 years (40%) and were males.The sequencing was analyzed for the type of mutation (in-frame deletions, substitutions, duplications), the nucleotide change, whether the mutation was a heterozygous or a homozygous mutation and whether this mutation was disease causing or not.

## Results

All cases of histologically and immunohistochemically proven GISTs diagnosed in the Department of Pathology of our tertiary teaching institution over a period of three years were included in the study. A total of 20 cases were identified.

**Figure 1:**
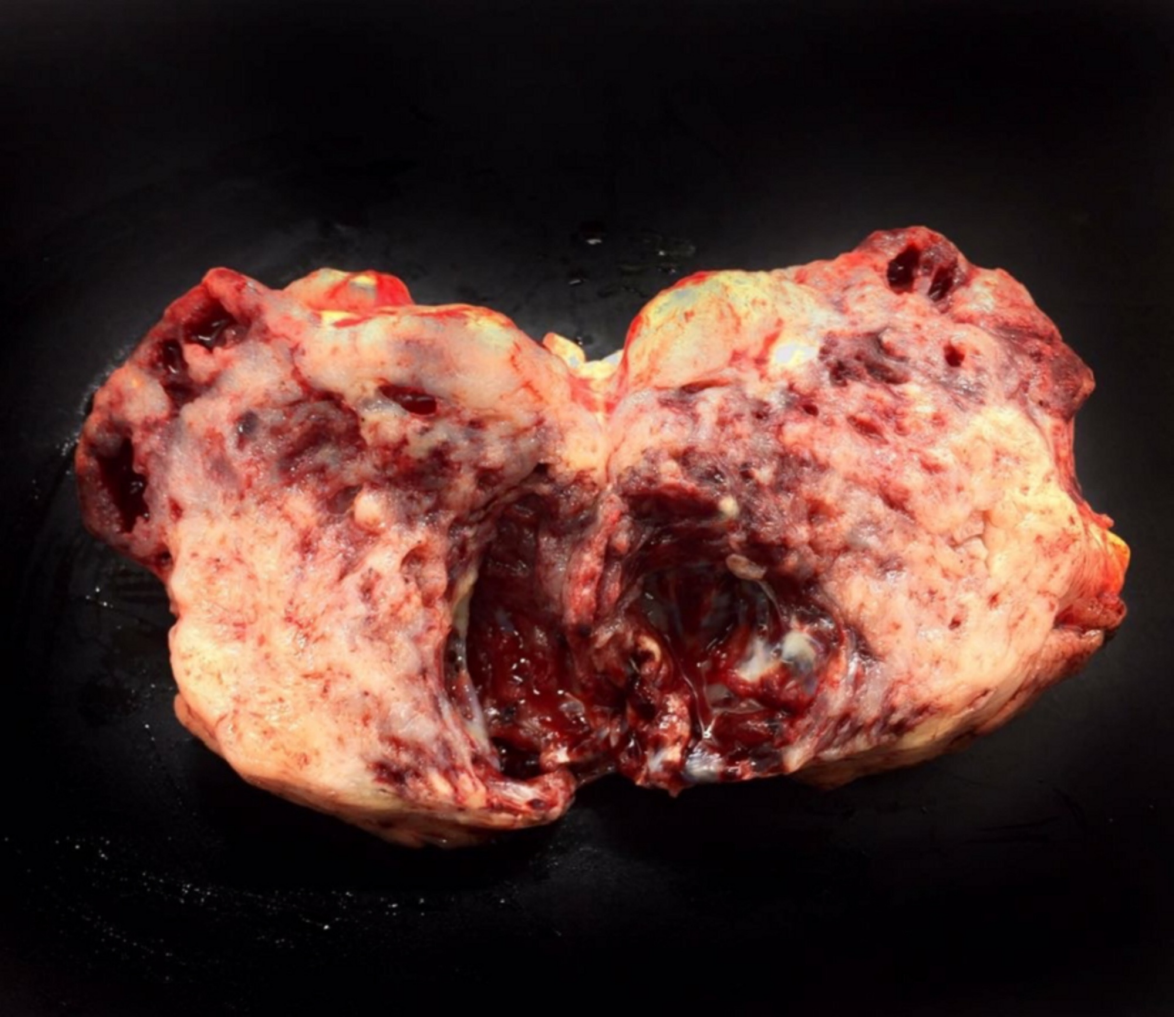
Gastric GIST with a Fleshy appearance and Central Degeneration

The age of study participants ranged from 35 to 68 years with 65% cases in men. Stomach was the most common site of the tumor (50%) and about 35% of the tumors were of the size of 5-9 centimeters. Grossly, the tumours were submucosal, circumscribed and unencapsulated. On sectioning, the cut surface varied in colour from grey white to grey brown, solid with few having cystic areas and some tumours had areas of necrosis (Figure 1).

**Figure 2:**
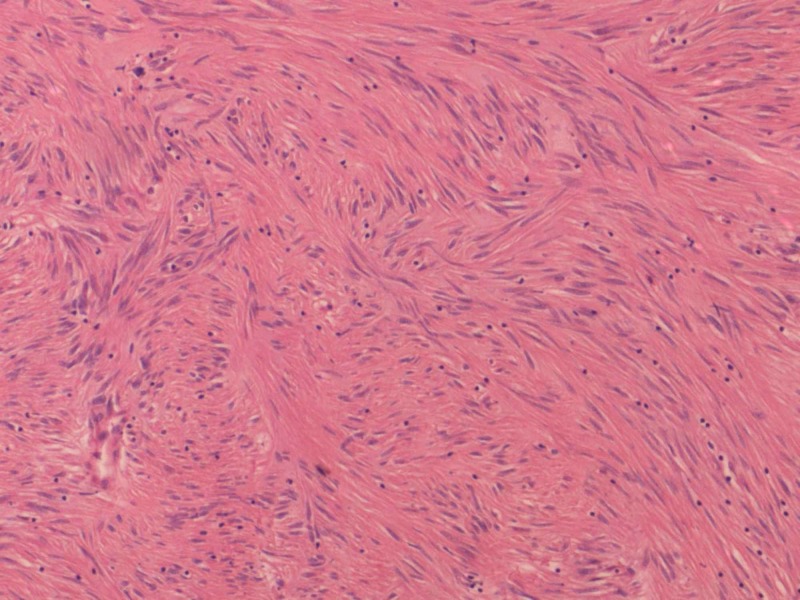
Microphotograph of Spindle Cell GIST in a Fascicular Pattern (H&E 40x)

**Figure 3:**
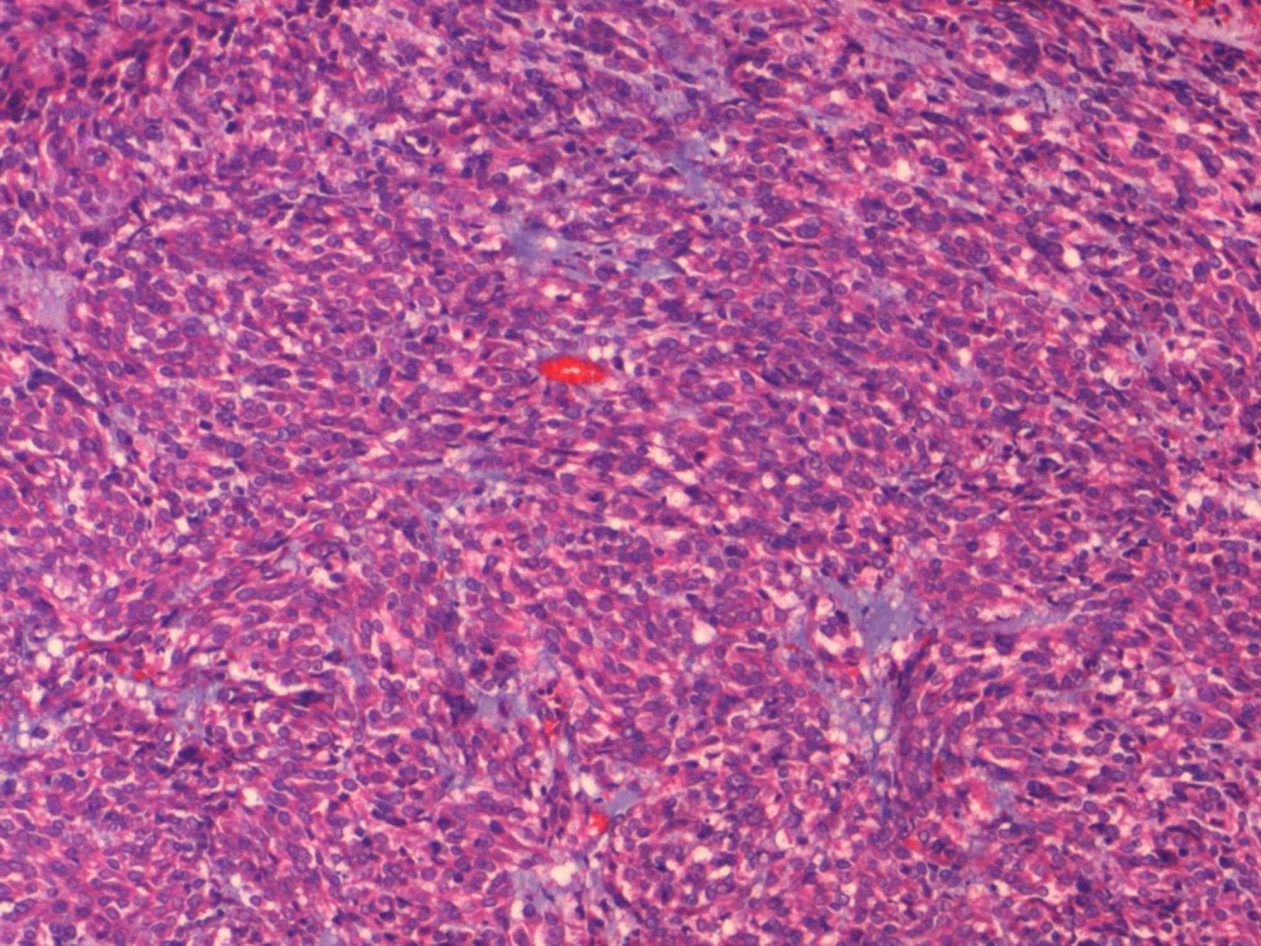
: Microphotograph of Epitheloid GIST with a Nested Growth Pattern (H&E 100x)

Microscopically, 15 tumours were classified histologically as spindle cell while four cases were classified as mixed type comprising of both spindle cell(Figure 2) and epitheloid (Figure 3) morphology. Epitheloid variant was found in one case only. The staging of the tumors revealed that 50% of the tumors belonged to pT4 stage, while 40% of them belonged to pT3 stage. Majority of the tumors were high risk (45%) followed by intermediate risk (30%).

**Figure 4:**
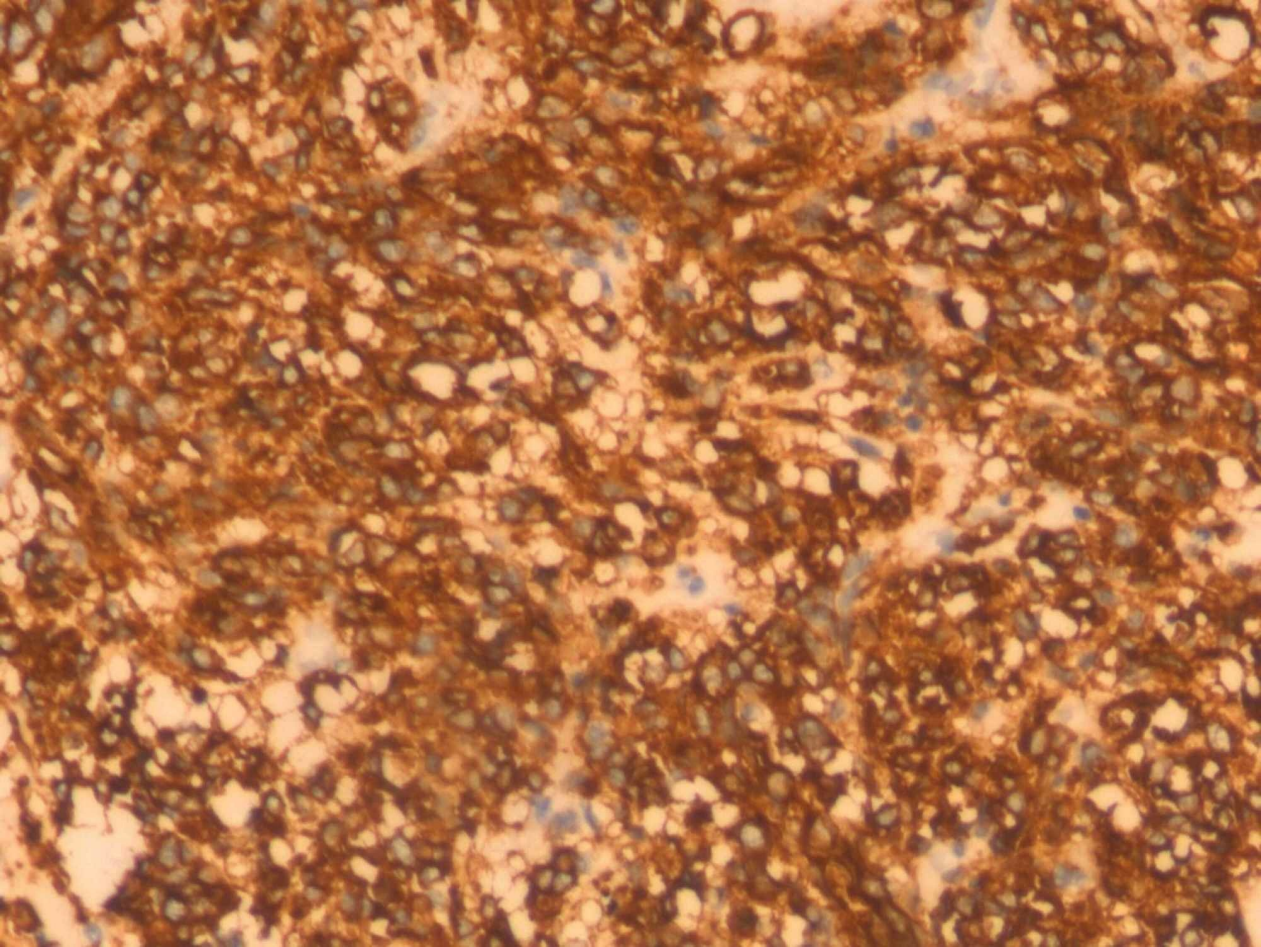
Microphotograph of C-KIT Immunoreactivity at high power (H&E 200x)

Immunohistochemistry for CD 117 was done in 20 cases. In 19 cases, strong and diffuse cytoplasmic positivity for CD 117 was seen (Figure 4). One case was negative for CD 117. However, this case showed a positive immunohistochemical staining for Discovered on GIST-1 (DOG1) and PDGFRA.

C-Kit mutation analysis

**Figure 5:**
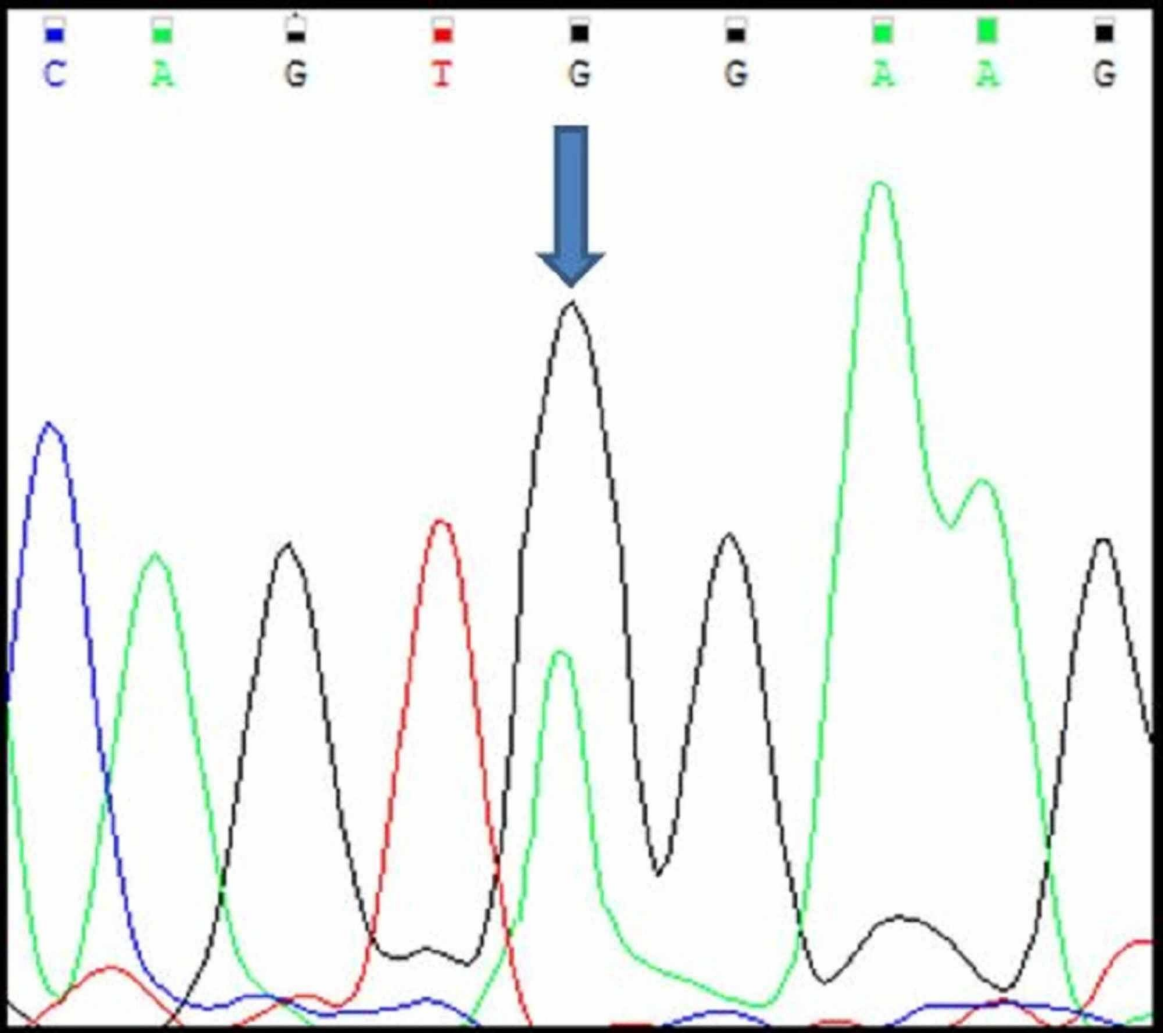
Case A1 KIT Exon 11 Sangers Sequence Change

**Figure 6:**
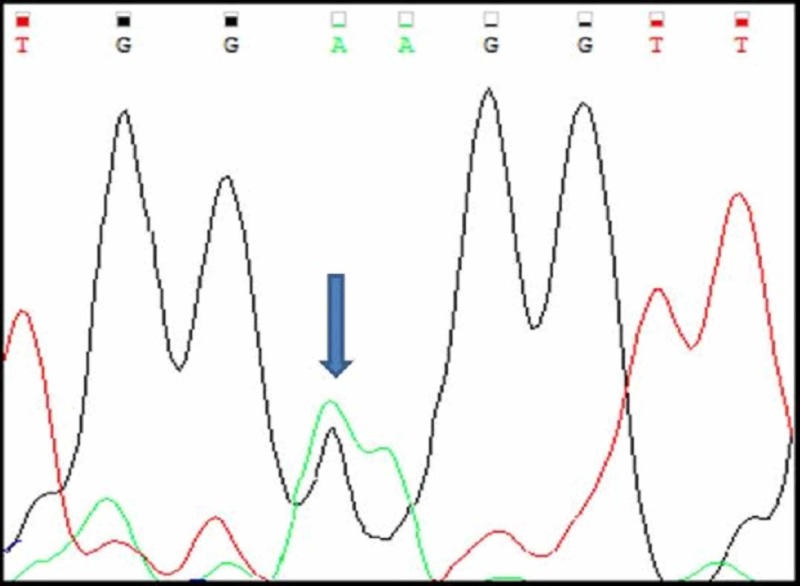
Case A2 KIT Exon 11 Sangers Sequence Change

**Figure 7:**
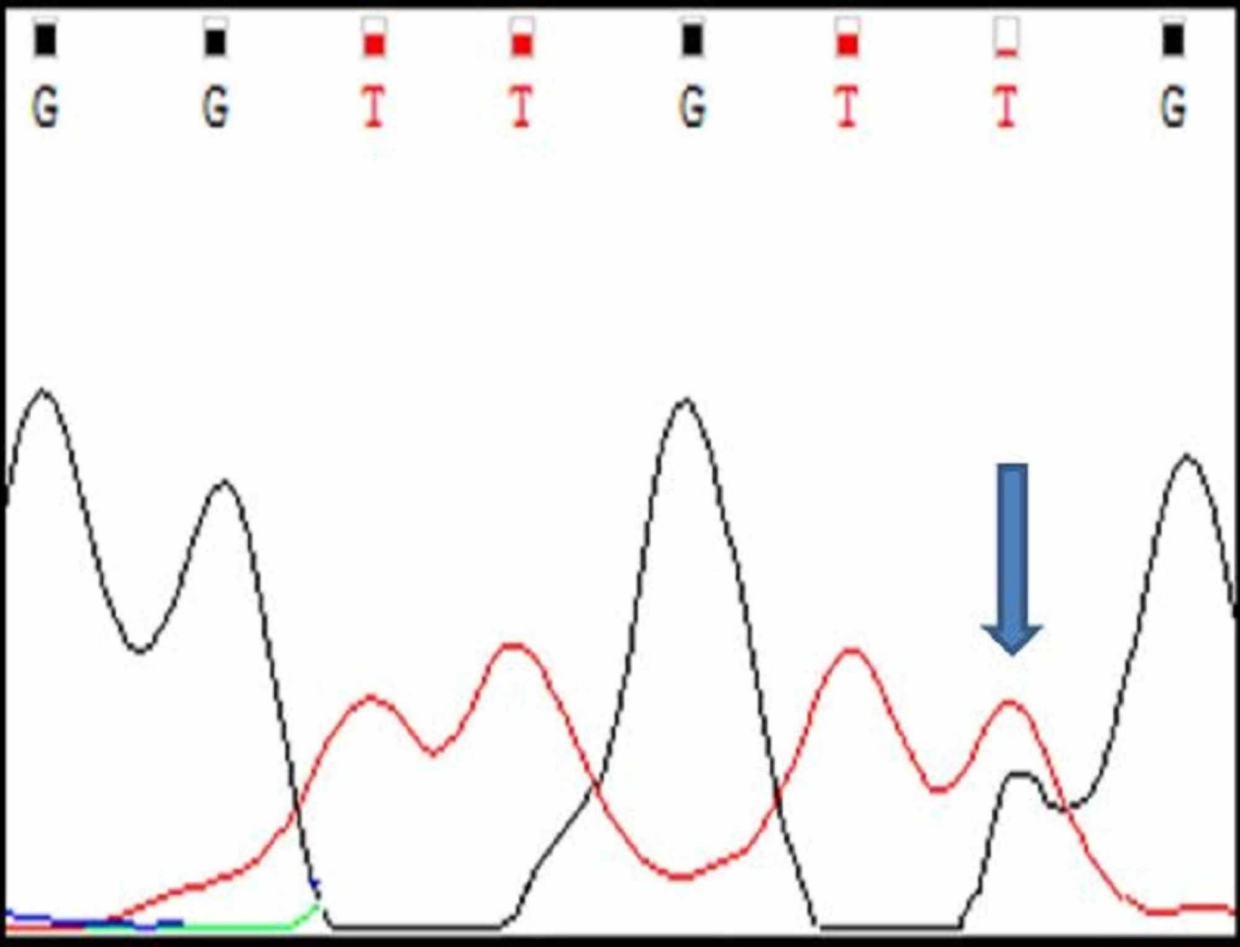
Case A3 KIT Exon 11 Sangers Sequence Change

**Figure 8:**
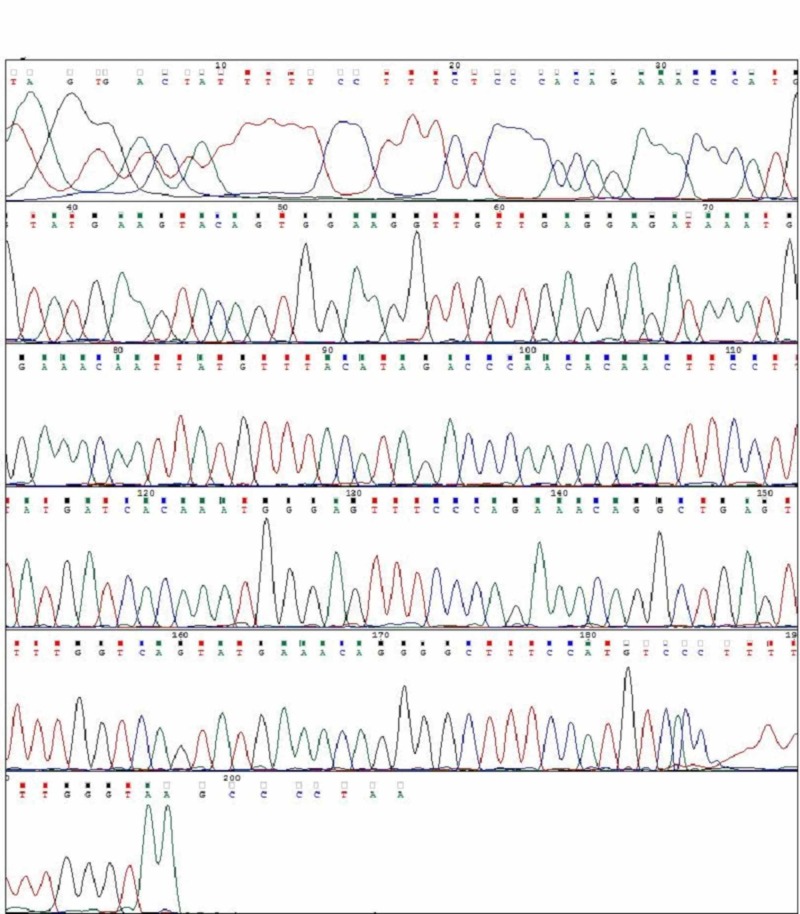
Exon 11 Normal Sangers Sequence In Non Mutated Cases

Of the 20 cases, three cases (A1, A2, A3) had mutations in c-KIT exon 11. All the three mutations were a single nucleotide substitution with heterozygous mutations. Their nucleotide change and amino acid sequence were analyzed and using mutation taster software, we predicted whether the change is disease causing or not (Figure 5-7; Table 2). The change in amino acid in case number A1 was from tryptophan at position 557 to stop codon (c.1670G>A), in case number A2 the change in amino acid was from lysine at position 558 to glutamic acid (c.1672A>G) and in case A3 there was no amino acid change but the nucleotide change identified was c.1680 T>G. The rest of the 17 tumours had a normal exon 11 sequence (Figure 8). Correlation with clinico-pathological findings shows that exon 11 substitution mutations occurred in the age group 58-61 years. They were present in different locations namely stomach, duodenum and jejunum. Histologically, all the three cases had a spindle cell morphology. The three tumours were classified as intermediate risk.

**Table 2:** Exon 11 KIT Mutations

CASE NUMBER	AGE	SEX	SITE	MORPHOLOGY	RISK STRATIFICATION	GENE	EXON	MUTATION STATUS	NUCLEOTIDE CHANGE	AMINO ACID CHANGE	PREDICTION (MUTATION TASTER)
A1	58yrs	Male	Duodenum	Spindle cell	Intermediate risk	KIT	11	Heterozygous	TGG-TAG (c.1670G>A)	Try557Stop codon	Disease causing
A2	60yrs	Female	Stomach	Spindle cell	Intermediate risk	KIT	11	Heterozygous	AAG-GAG (c.1672A>G)	Lys558Glu	Disease causing
A3	61yrs	Male	Jejunum	Spindle cell	Intermediate risk	KIT	11	Heterozygous	GTT-GTG (c.1680 T>G)	Val-Val (no amino acid change)	Disease causing

**Figure 9:**
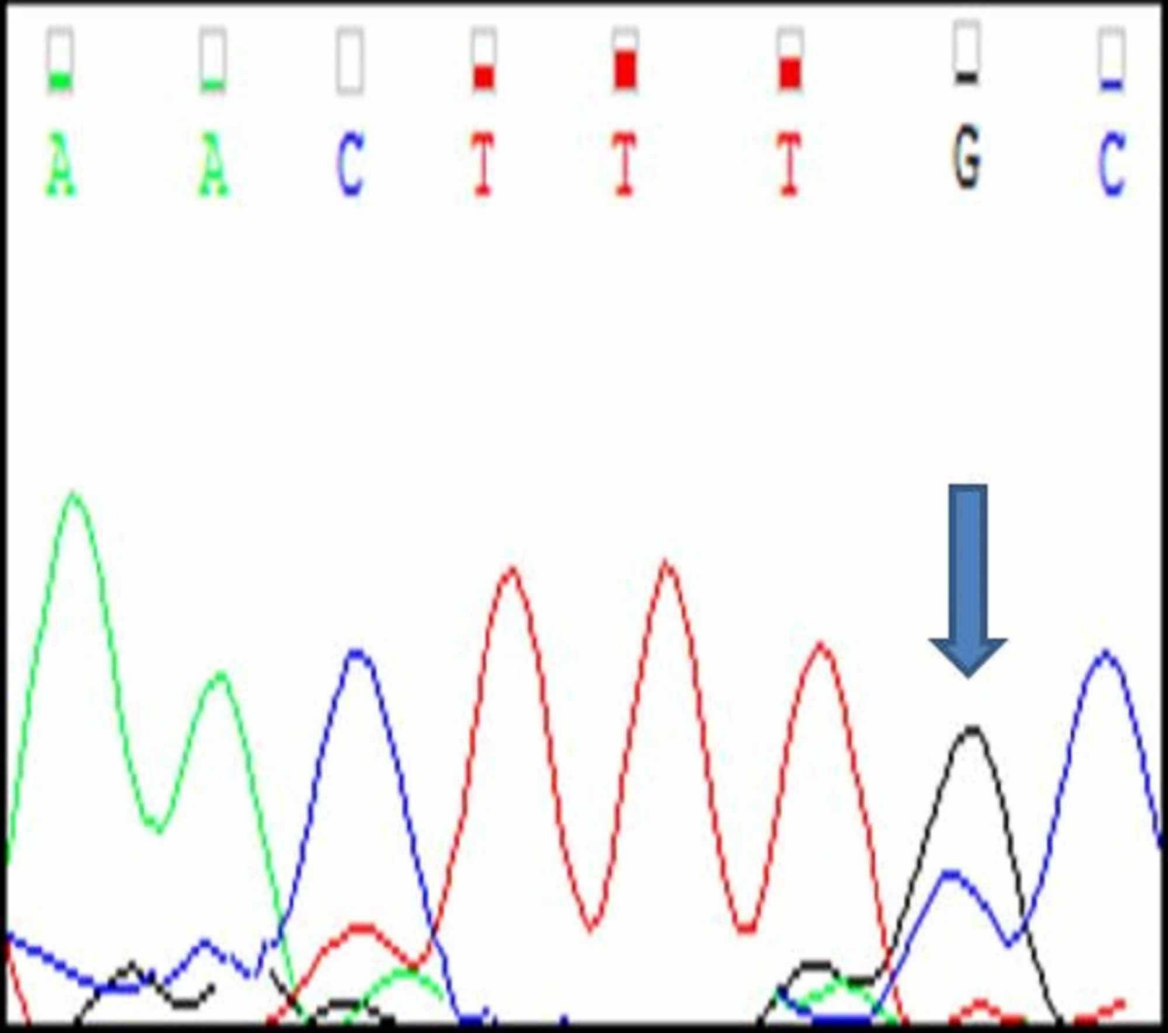
Case A4 KIT Exon 9 Sangers Sequence Change

**Figure 10:**
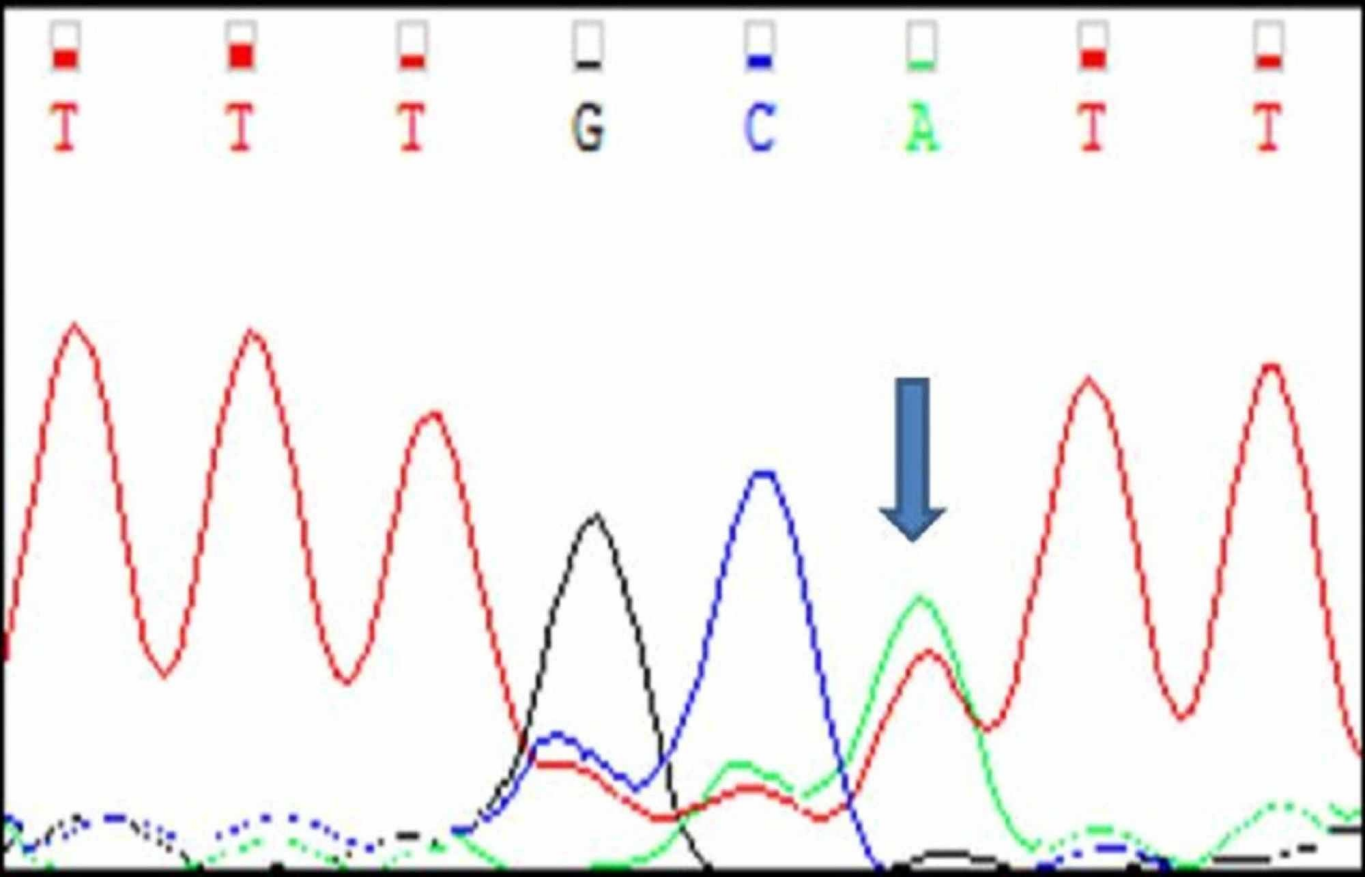
Case A5 KIT Exon 9 Sangers Sequence Change

**Figure 11:**
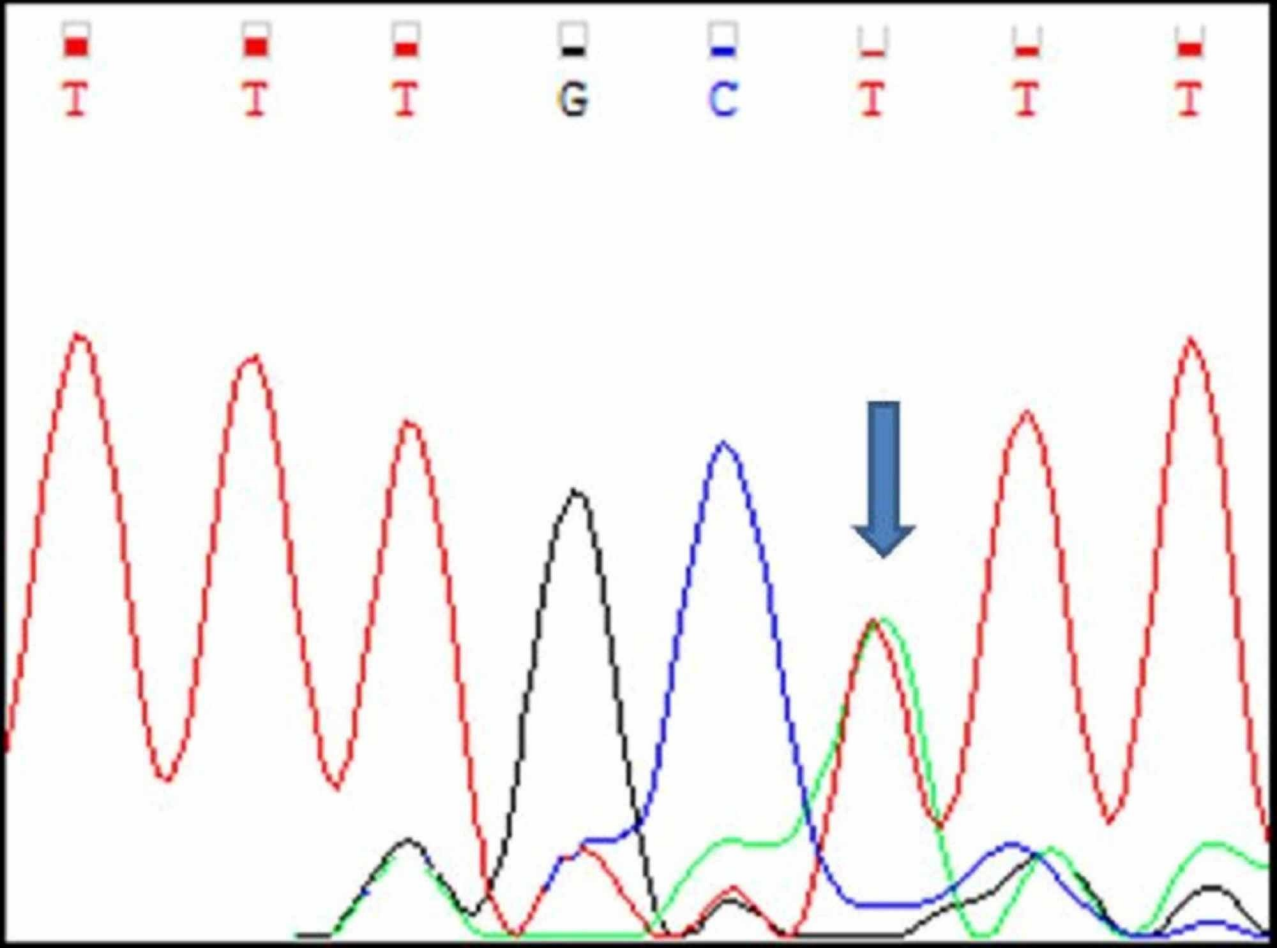
Case A6 KIT Exon 9 Sangers Sequence Change

**Figure 12:**
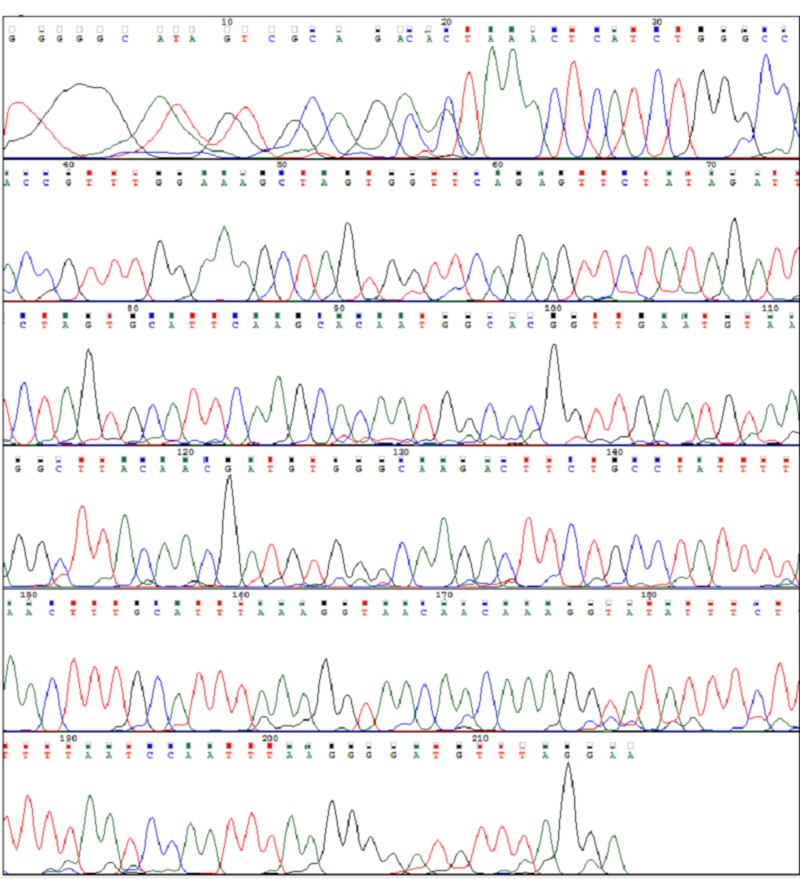
: Exon 9 Normal Sangers Sequence in Non Mutated Cases

Three cases (A4, A5, A6) had mutations in c-KIT exon 9. All the three cases were heterozygous mutations with single nucleotide substitution. Their nucleotide change and amino acid sequence were analyzed and using mutation taster software, we predicted whether the change is disease causing or not (Figure 9-11; Table 3). The change in amino acid in case number A4 was from alanine at position 507 to proline (c.1519G>C). In A5 and A6 there was no change in the aminoacid but nucleotide change was identified as c.1521A>T. Rest of the 17 tumours had normal exon 9 sequence (Figure 12). Correlation with clinicopathological findings shows that Exon 9 mutations occurred in the age group 45 -60 years and the tumours occurred in different locations namely stomach, jejunum and ileum but predominantly in the small intestine. Histologically, two cases had spindle cell morphology and one case had a mixed pattern of epitheloid and spindle cell morphology. Two tumours were classified as intermediate risk and one tumour as high risk.

**Table 3:** Exon 9 KIT Mutations

CASE NUMBER	AGE	SEX	SITE	MORPHOLOGY	RISK STRATIFICATION	GENE	EXON	MUTATION STATUS	NUCLEOTIDE CHANGE	AMINO ACID CHANGE	PREDICTION (MUTATION TASTER)
A4	52yrs	Female	Jejunum	Spindle cell	Intermediate risk	KIT	9	Heterozygous	GCA-CCA (c.1519G>c)	Ala507Pro	Disease causing
A5	60yrs	Female	Ileum	Mixed	High risk	KIT	9	Heterozygous	GCA-GCT (c.1521A>T)	Ala-Ala (no amino acid change)	Disease causing
A6	45yrs	Male	Stomach	Spindle cell	Intermediate risk	KIT	9	Heterozygous	GCA-GCT (c.1521A>T)	Ala-Ala (no amino acid change)	Disease causing

BRAF V600E mutation analysis

**Figure 13:**
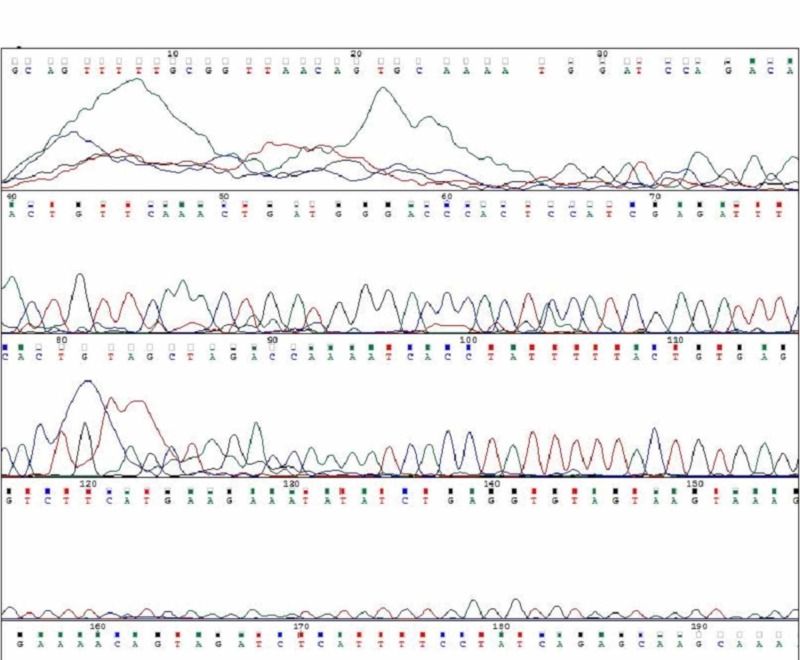
Normal BRAF V600E Sangers Sequence in Non Mutated Cases

**Figure 14:**
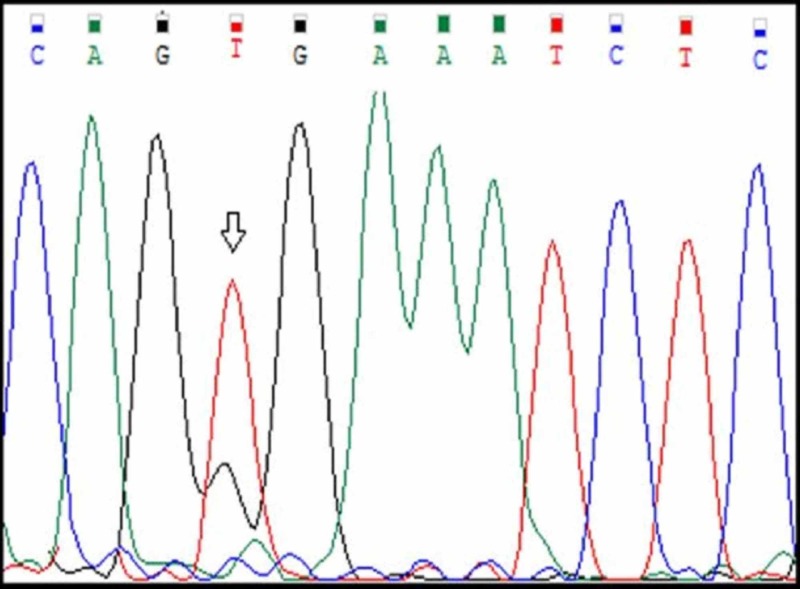
Case A7 BRAF V600E Sangers Sequence Change

Among the 20 cases, 19 cases had normal BRAF sequence (Figure 13) and only one tumour (Case number A7) had BRAF V600E mutational sequence (Figure 14, Table 4). A single nucleotide substitution was identified (c.1799T>G) from Valine to glycine at position 600 (c.1799T>G). The clinicopathological correlation revealed that the single case was identified in a 58 years old male patient and the tumour was located in the jejunum. The size of the tumour was 15cm. Histologically, the tumour was composed of a mixed pattern of epitheloid and spindle cell morphology and had a mitotic rate of 12/10HPF. The tumour was classified as high risk.

 

**Table 4:** BRAFV600E Mutation

CASE NUMBER	AGE	SEX	SITE	MORPHOLOGY	RISK STRATIFICATION	GENE	EXON	MUTATION STATUS	NUCLEOTIDE CHANGE	AMINO ACID CHANGE	PREDICTION (MUTATION TASTER)
A7	58yrs	Male	Jejunum	Mixed	High risk	BRAF	15	Heterozygous	GTG-GGG (c.1799T>G)	Val(600)Gly	Disease causing

## Discussion

The mechanistic basis of GIST is based on multiple genetic alterations. Abnormalities in C-KIT (exon 9, 11, 13 and 17), PDGFRA, BRAFV600E, SDH (A,B,C) and NF1 genes have been implicated in the pathogenesis of GIST. The present study was carried out to evaluate the nature of expression of c-KIT exon 9, 11 and BRAF V600E genetic mutations and their alterations at the molecular level. The mean age of presentation was 54 years with maximum cases seen between 50-59 years (40%). Similar findings were seen in a study done by Bhalgami et al. [6]. Moreover, the peak incidence of GIST was seen among the men (65%) compared to the women (35%) in the present study. This finding was comparable with a study done by DeMatteo et al. [7]. The most common site of presentation of the tumor in the GI tract was stomach (50%) followed by jejunum and ileum. This was in accordance with a study done by Miettinen et al. and Gomez et al. [8,9].

The most common histological type of the GIST was spindle cell type, which was majorly seen in the small intestine. Mixed type was equally present in stomach and small intestine. Among the spindle cell type, the growth pattern commonly seen was fascicular. Epithelioid type was seen in the stomach tumors. Large sized tumors (>10 cm) showed increased mitotic rate. This finding was in accordance with studies done by Vij et al. and Roggen et al. [10,11]. In the present study, 45% of the tumors were categorized as high risk of which majority of the tumors occurred in the stomach. The risk stratification was carried out in accordance to the studies done by Miettinen et al. [12,13]. Moreover, 95% of the specimens morphologically designated as GIST stained positively for CD 117. Based on a study done by Medeiros et al., it is acceptable that all GISTs are not positive for CD117 as these negative tumors may harbor other mutations in other genes such as PDGFRA [14].

The biological mechanism of GIST in the knowledge of c-KIT mutations was first unraveled by Hirota et al. in 1998 [15]. Similar observations were later seen in studies by Rubin et al. and Heinrich et al., where activating mutations on the tyrosine kinase receptor were considered to be crucial in the pathogenesis of GIST [16,17]. In their studies, Colucci et al., Vosseller et al. and Kapur et al. found that signaling pathways activated by KIT oncoproteins modify crucial aspects of the GIST cell phenotype. The GIST signaling pathways differ from those in the non-neoplastic cells, and depending on the location and nature of the mutation, the exact signaling mechanism also varies between GIST tumors. Critical clinicopathological and biological parameters including GIST apoptotic activity, mitotic activity, metastatic capability and imatinib response are all influenced by c-KIT structure and sequence [18-20].

In a study of 100 cases by Pai et al. in the Indian population, exon 11 mutations were identified in 57% of cases. In-frame deletions in 35 cases, 11 substitutions cases, nine cases of double mutations, one case each of insertion and duplication. The most common mutation was tryptophan557_lysine558 del (c.1669_1674delTGGAAG) in 13 cases. The substitution mutations were Val559Asp, Val560Asp, Val559Ala, Val560Gly, Thr574Ile and Leu576Pro; among these nine were homozygous and two were heterozygous [21]. This was not in concordance with our study as we reported different set of missense mutations. In the present study, c-KIT exon 11 mutations were identified in three cases of GISTs. All the three cases had single nucleotide substitution and the missense mutations identified were Trp557Stop (c.1670G>A), Lys558Glu (c.1672A>G), Val-Val (c.1680T>G). All the three cases were heterozygous mutations.

In our study the three C-KIT exon 11 mutations occurred in the stomach, duodenum and jejunum. This was in concordance with a study by Lasota et al. where the exon 11 mutations were seen in different sites including gastric, small intestinal and rectal tumours [22].

In their study of 87 patients, Daniels et al. found in-frame deletions, single nucleotide substitutions and duplications in C-KIT exon 11. In their study all the deletions were classified as high risk, whereas substitution and duplication mutations were classified as intermediate risk [23]. This was in accordance with our study where all the three C-KIT exon 11 mutations were classified as intermediate risk.

In a study by Pai et al., out of 100 cases 10 cases were identified to harbor exon 9 mutations. All the 10 cases had duplications in Alanine to tyrosine at codons 502 -503. Nine tumours were present in the small intestine and one in the retroperitoneum. All the tumours had a spindle cell morphology [21]. Lux et al. and Lasota et al. in their studies found heterozygous exon 9 mutations in GISTs. All the tumours were localized to the small intestine. The most common morphology identified was spindle cell morphology. The mutation seen in these cases were all duplications. The most common duplications were alanine at position 502 and tyrosine at position 503. These mutations activate the receptor via a ligand independent oligomerization [24,25]. These studies were not in concordance with our study where there were single nucleotide substitutions rather than deletions. In our study, the mutations identified were Ala507Pro (c.1519G>C) and two cases had no amino acid change with nucleotide substitution Ala -Ala (c.1521A>T). The site of occurrence of tumour was small intestine (two cases) and stomach (one case). The most common histology was spindle cell type and one mixed type. The reported exon 9 mutations in the present work clearly differs from the prototypical c-KIT exon 9 mutated GIST.

A single case of GIST in stomach with mixed morphology was studied by Grabellus et al., wherein a c-KIT exon 9 single nucleotide substitution Ser476Ile (c.1472G>T) was observed [26]. This patient had a progression free survival of 15 months, since the median progression free survival of usual c-KIT exon 9 mutations is less than 1 year under imatinib standard therapy. The mutation identified was similar to our study. However, the nucleotide substitution was not similar.

BRAF V600E mutations in GISTs were first demonstrated by Agaram et al. in a series of wild type GISTs. BRAF mutations in their study occurred in the small intestine [4]. Our study was in concordance with this study where the site of tumour was in the jejunum. Hostein et al. detected BRAF mutations in 13% of the wild-type GISTs and they identified that the mutation can manifest in any histological pattern including spindle cell, epitheloid and mixed morphology of spindle cell and epitheloid type [27]. This was in concordance with our study where the tumors had a mixed morphology. Agaram et al. demonstrated that these BRAF mutated GISTs are usually classified as high risk tumours. This was also in concordance with our study where the tumour was classified as high risk [4].

Patil et al. demonstrated that mutations in BRAF on exon 15 were a DNA base substitution of thymine for adenine (T to A) that converts valine to glutamic acid of amino acid residue 600 (BRAF V600E) [28]. In the present study, heterozygous mutations were identified in nucleotidec.1799 T>G at exon 15 where there was a substitution of valine to glycine at position 600 (Val600Gly). Though the exon 15 position of mutation (Val600) was in concordance with our study, the nucleotide change was not similar.

In studies by Falchook et al. and Agaram et al., in the therapy for BRAF mutant GISTs, they found that the patient did not respond to a normal dose of imatinib and also required higher doses of imatinib or sorafinib therapy [29,4]. However at present our patient is doing well with the standard doses of imatinib.

Hence, in view of varied clinical outcomes, molecular studies of GISTs is very essential for understanding the pathogenesis, the behavior pattern, designing the spectrum of targeted therapies and assessing recurrence. Primary diagnosis of GISTs is based on histopathology and immunohistochemistry. However, with rapidly evolving personalized therapy, evaluation of mutation will become essential to assess prognosis and design targeted therapies. This study provides an interesting and limited insight in identifying the molecular changes in GISTs at the exonic level using DNA based sanger sequencing.

## Conclusions

Molecular pathways play a significant role in determining the pathogenesis and progression of GIST. The c-KIT exon 9 and 11 mutation together constituted 30% of the mutations in our limited series and majority of them were either categorized as intermediate or high risk. One case with BRAFV600E mutation was identified in high risk category. Therefore, with the evolution of personalized therapies for malignancies, assessment of mutations and molecular studies become increasingly valid and essential for both diagnosis and prognosis.
